# Oridonin Attenuates Lipopolysaccharide-Induced ROS Accumulation and Inflammation in HK-2 Cells

**DOI:** 10.1155/2020/9724520

**Published:** 2020-02-27

**Authors:** Jen-Hsuan Huang, Chou-Chin Lan, Ya-Ting Hsu, Cheng-Lin Tsai, I-Shiang Tzeng, Po Wang, Chan-Yen Kuo, Po-Chun Hsieh

**Affiliations:** ^1^Department of Anesthesiology, Show Chwan Memorial Hospital, Changhua, Taiwan; ^2^Division of Pulmonary Medicine, Taipei Tzu Chi Hospital, Buddhist Tzu Chi Medical Foundation, New Taipei, Taiwan; ^3^School of Medicine, Tzu Chi University, Hualien, Taiwan; ^4^Department of Chinese Medicine, Taipei Tzu Chi Hospital, Buddhist Tzu Chi Medical Foundation, New Taipei City, Taiwan; ^5^Department of Research, Taipei Tzu Chi Hospital, Buddhist Tzu Chi Medical Foundation, New Taipei City, Taiwan

## Abstract

Renal tubulointerstitial inflammation plays an important role in chronic kidney disease (CKD). Inflammation reduction is a good strategy to combat CKD. Oridonin, an ent-kaurane diterpenoid isolated from *Rabdosia rubescens* (Donglingcao), is considered as an effective natural candidate for the treatment of anti-inflammatory, antiviral, and antibacterial activities, including liver fibrosis and many tumors; however, no study has demonstrated its effect on lipopolysaccharide- (LPS-) induced renal inflammation. To investigate the anti-inflammatory effects of oridonin on human renal proximal tubular epithelial cells (HK-2 cells), the expression levels of c-Jun N-terminal kinase (JNK) and reactive oxygen species (ROS) were evaluated by Western blot analysis and 2′,7′-dichlorofluorescein diacetate (DCF-DA) staining, respectively. The level of intracellular ROS increased in a dose-dependent manner following LPS treatment, whereas oridonin inhibited this effect, suggestive of its ability to prevent ROS accumulation. As the mitogen-activated protein kinase (MAPK) family of enzymes plays an important role in physiological responses, we examined the activation of JNK by Western blotting and found that oridonin attenuated LPS-induced JNK phosphorylation. Oridonin also attenuated RAW 264.7 cell chemotaxis towards LPS-treated HK-2 cells. Taken together, oridonin protected against LPS-induced inflammation including ROS accumulation, JNK activation, NF-*κ*B nuclear translocation in HK-2 cells, and functionally blocked macrophage chemotaxis towards LPS-treated HK-2 cells. Oridonin may exhibit therapeutic potential by the anti-inflammation effect in LPS-treated HK-2 cells.

## 1. Introduction

Chronic kidney disease (CKD) is characterized by a progressive and irreversible exacerbation of the renal excretory function that necessitates renal replacement therapy in the form of dialysis or renal transplants and may also lead to death [1]. Therefore, CKD is a global health problem with serious implications. Renal tubulointerstitial inflammation has an important role in fibrosis, which is the key pathogenetic alteration associated with CKD [2].

Wang et al. suggested that an increase in oxidative stress plays a critical role in mediating metabolic syndrome-induced tubulointerstitial injury [3]. Albuminuria may serve as an endogenous danger-associated molecular pattern (DAMP) that stimulates tubulointerstitial inflammation via mitochondrial reactive oxygen species- (mtROS-) mediated activation of the cytoplasmic Nlrp3 inflammasome in HK-2 cells [4]. Evidence indicates that SIRT3, an antiaging molecule regulated by calorie restriction and mitochondrion-localized NAD (+) -dependent deacetylase, positively regulates both mitochondrial oxidative capacity and antioxidant gene expression, thereby reducing ROS accumulation in mouse proximal tubular cells [5]. It is well known that activated macrophages respond to inflammation induced by bacterial lipopolysaccharide (LPS) by producing cytokines (interferon-gamma, granulocyte-monocyte colony-stimulating factor, and tumor necrosis factor-alpha), extracellular matrix proteins, and other chemical mediators [6]. LPS plays a crucial role in macrophage infiltration and progressive chronic kidney inflammatory injury and fibrosis via activation of the mammalian target of rapamycin- (mTOR-) dependent pathway [7]. However, the pharmacological effects of oridonin and the underlying mechanism of action in renal tubulointerstitial inflammation are still unknown.

Oridonin, an ent-kaurane diterpenoid isolated from *Rabdosia rubescens* (Donglingcao), has various pharmacological and biological properties such as anti-inflammatory, antiviral, and antibacterial activities [8]. Many studies have suggested that oridonin has potent anticancer activity against several cancers, including lung cancer, colorectal cancer, and hepatocellular carcinoma, *in vitro* and *in vivo* [9–12] and may exert beneficial effect against Alzheimer's disease [13], acute lung injury [14], postinflammatory irritable bowel syndrome [15], and Crohn's disease [16]. These diseases are closely related to inflammation. It is known that mtROS are signaling molecules involved in the physiological recruitment of patrolling cells and pathological recruitment of inflammatory cells [17]. ROS accumulation ultimately leads to DNA damage and cell death via glutathione depletion and caspase-3 activation [18, 19]. Therefore, it is important to identify the candidate compounds that respond to the inflammation caused by ROS overproduction during the development of renal tubulointerstitial disease.

Macrophages are important tumor-infiltrating cells and play critical roles in tumorigenesis and metastasis [20]. Liu et al. reported that LPS induces chemokines secretions, including interleukin-8 (IL-8), monocyte chemoattractant protein-1 (MCP-1), and macrophage inflammatory protein-1*α* (MIP-1*α*) [21]. Therefore, a survey of candidate compounds with potential antichemotaxis effects on RAW 264.7 cell, as reported by Kuo et al. in a previous study, is needed [22].

This is the first study aiming for investigating the anti-inflammatory effects of oridonin on LPS stimulated HK-2 cells and the underlying mechanisms of action, including ROS accumulation, c-Jun N-terminal kinase (JNK) phosphorylation, nuclear factor kappa B (NF-*κ*B) nuclear translocation, and RAW 264.7 cell chemotaxis.

## 2. Materials and Methods

### 2.1. Reagent

Oridonin (CAS 28957-04-2) was purchased from Merck (MO, USA). The HPLC description was ≥93%.

### 2.2. Antibodies

The following antibodies were used for immunofluorescence staining and Western blotting: rabbit polyclonal antibodies to p-JNK (ABclonal, MA, USA), JNK (ABclonal, MA, USA), *β*-actin (ABclonal, MA, USA), p65 (Cell signaling, MA, USA), fibrillarin (Cell signaling, MA, USA), Cox-2 (ABclonal, MA, USA), iNOS (Abcam, UK), CTGF (ABclonal, MA, USA), and *a*-SMA (ABclonal, MA, USA).

### 2.3. Cell Culture

Human renal proximal tubular epithelial cells (HK-2) were purchased from Bioresource Collection and Research Center (BCRC, Taiwan) and cultured in T-75 flasks (Corning, NY, USA) in Dulbecco's modified Eagle's medium (DMEM; Ham's F12 (Gibco, NY, USA) supplemented with 10% fetal bovine serum (FBS), 25 mM D-glucose, 2 mM L-glutamine, 1 mM sodium pyruvate, and penicillin-streptomycin (50 U/mL; Sigma, MO, USA) at 37°C in 5% CO_2_/95% air. The culture medium was replaced with fresh medium on alternate days. The cells were trypsinized once they reached 70% confluence and used for subsequent experiments.

The murine monocyte/macrophage cell line RAW 264.7 (BCRC #60001) was purchased from BCRC (Taiwan). RAW 264.7 cell line was cultured in DMEM supplemented with 10% FBS, 4 mM L-glutamine, 4,500 mg/L glucose, 1 mM sodium pyruvate, 1,500 mg/L sodium bicarbonate, and 50 U/mL penicillin-streptomycin (Sigma-Aldrich, St. Louis, MO, USA) at 37°C in 5% CO_2_/95% air for no more than five passages. The culture medium was replaced on alternate days. The cells were collected via trypsinization after reaching 50%–60% confluence and used for subsequent experiments.

### 2.4. Western Blot Analysis

The Western blot analysis was according to our previous studies with some modifications [23]. Total proteins were extracted from HK-2 cells using ice-cold radioimmunoprecipitation assay (RIPA) buffer (20 mM Tris-HCl [pH 7.4], 150 mM sodium chloride (NaCl), 1 mM EGTA, 1 mM sodium fluoride (NaF)), 2 mM sodium orthovanadate (Na_3_VO_4_), 1 mM phenylmethylsulphonyl fluoride, 1% dilution of Sigma protease cocktail, and 1% Triton X-100). Extracted proteins were separated by 10% or 15% sodium dodecyl sulfate-polyacrylamide gel electrophoresis (SDS-PAGE), and the separated protein bands were transferred onto nitrocellulose membranes. Immunoblotting was performed using specific primary antibodies and horseradish peroxidase-conjugated secondary antibodies (Cell Signaling Technology, MA, USA). Peroxidase activity was assessed using an enhanced chemiluminescence detection kit (PerkinElmer Life Science, MA, USA).

### 2.5. Measurement of Intracellular ROS Generation

The analysis of the measurement of intracellular ROS generation was according to our previous studies with some modifications [23]. Cells were washed with phosphate-buffered saline (PBS) and incubated with 10 *μ*M of 2′,7′-dichlorofluorescein diacetate (DCF-DA; Sigma, MO, USA) at 37°C for 30 min in the dark. In the presence of ROS, DCF-DA is oxidized to produce fluorescence. After incubation, the cells were trypsinized and washed thrice with ice-cold PBS. The ROS level was quantified by flow cytometry (BD Biosciences, CA, USA) using 488 nm excitation/585 nm emission filters.

### 2.6. Cell Viability Assay

We used WST-1 assay (Abcam, UK) to detect the cell viability, according to the manufacturer's instructions. In brief, HK-2 cells were seeded at a density of 5 × 10^4^ cells/ml in 24-well plates and cultured in phenol red-free DMEM containing 0.5% heat-inactivated FBS for 24 h. After incubation, cells were incubated with indicated concentrations of LPS for 24 h. The WST-1 reagent was then added to the medium and incubated at 37°C for 2 h. The absorbance was measured at 450 nm in a microplate reader (BIO-RAD, CA, USA).

### 2.7. Chemotaxis Assay

The chemotaxis analysis was according to our previous studies with some modifications [23]. We used 24-well Transwell plates (8 *μ*m pore size; Corning, NY, USA) to measure chemotaxis of RAW 264.7 cells. HK-2 cells were preconditioned with or without 30 *μ*M oridonin for 12 h before incubation with 1, 3, or 10 *μ*g/mL of LPS for 24 h. These cells were seeded into the lower chamber for 24 h in serum-free media supplemented with 5% bovine serum albumin (Sigma-Aldrich, MO, USA). RAW 264.7 cells (1 × 10^5^) were seeded in the upper chamber and placed over the lower chamber containing treated or untreated HK-2 cells. The upper chamber containing RAW 264.7 cells was used for the chemotaxis assay. The migratory cells on the lower side of the membrane were stained with 0.1% crystal violet for 5 min and washed with water; the membrane was scanned using EPSON V750 PRO scanner. Crystal violet was destained with methanol for 15 min, and the absorbance values were measured at OD570. All measured values were detected by Synergy HT (BioTek, VT, USA).

### 2.8. Nuclear Fraction Extraction

The nuclear fraction extraction was according to our previous studies with some modifications [23]. The nuclear fraction was extracted from HK-2 cells. The cells were collected and resuspended in a hypotonic buffer (10 mM HEPES, pH 7.9, 10 mM) potassium chloride (KCl), 1.5 mM magnesium chloride (MgCl_2_), 0.2 mM phenylmethylsulphonyl fluoride (PMSF), 20 *μ*g/mL aprotinin, 0.5 mM dithiothreitol (DTT), and 0.5% NP-40 on ice for 15 min. After centrifugation at 6,000 × *g* for 15 min at 4°C, the pellet was collected and washed with a basal buffer (hypotonic buffer without 0.5% NP-40). The cells were centrifuged at 6,000 × *g* for 15 min at 4°C, and the pellet was collected and resuspended in a hypertonic buffer (20 mM HEPES, pH 7.9, 400 mM KCl, 1.5 mM MgCl_2_, 0.2 mM PMSF, 20 *μ*g/mL aprotinin, 0.5 mM DTT, 0.2 mM ethylenediaminetetraacetic acid (EDTA), and 10% glycerol) at 25°C for 30 min. After centrifugation at 10,000 × *g* for 30 min at 4°C, the nuclear fraction in the supernatant was collected.

### 2.9. Statistical Analysis

Statistical analysis was performed with IBM SPSS Statistics 25 (IBM, NY, USA). Data are expressed as means ± standard deviation. Groups were compared with one-way or two-way analysis of variance (ANOVA) followed by Bonferroni post hoc analysis. A value of *p* < 0.05 was considered to indicate statistical significance.

## 3. Results

### 3.1. LPS-Induced ROS Accumulation, JNK Phosphorylation, and NF-*κ*B Nuclear Translocation in HK-2 Cells

**Figure 1:**
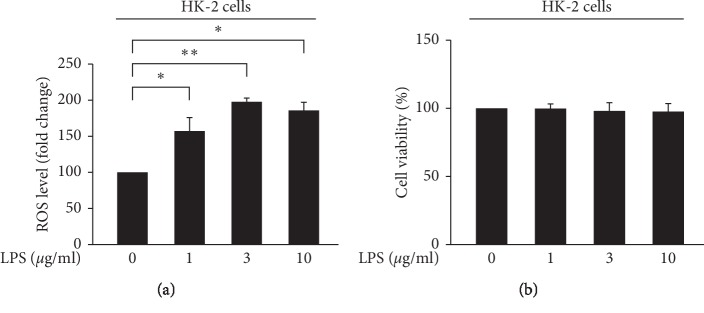
LPS-induced ROS accumulation. (a) HK-2 cells were treated with either the control or indicated concentrations of LPS for 24 h The levels of intracellular ROS were determined using DCF-DA, while the fluorescence signal was detected using a FACSCalibur instrument. (b) After the incubation period, cell viability was determined using WST-1 assay. All data are presented as the mean ± SD. *n* = 3. ^*∗*^*p* < 0.05, ^*∗∗*^*p* < 0.01.

**Figure 2:**
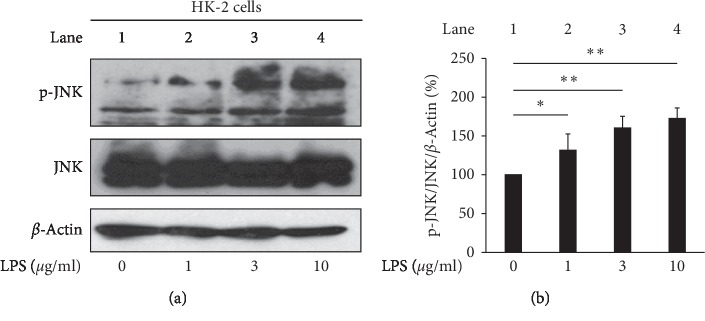
LPS-induced phosphorylation of JNK. (a) LPS-induced phosphorylation of JNK in a dose-dependent manner. HK-2 cells were treated with LPS at the indicated concentrations for 24 h. Phosphorylation of JNK was analyzed using immunoblotting analysis with antibodies against phosphorylated and total protein. (b) Densitometric analysis of all samples normalized against the level of total protein. All data are presented as mean ± SD. *n* = 3. ^*∗*^*p* < 0.05, ^*∗∗*^*p* < 0.01.

**Figure 3:**
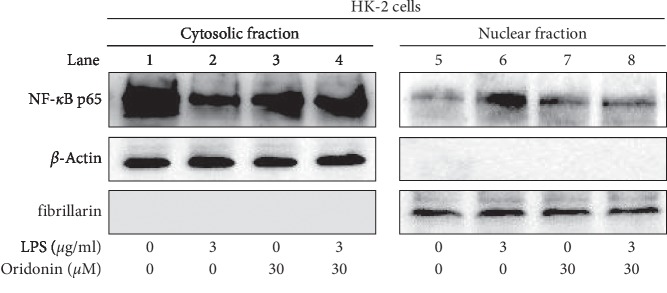
Oridonin attenuated LPS-induced NF-*κ*B nuclear translocation. HK-2 cells cultured and treated as above were collected, and cytosolic and nuclear fractions were isolated as described in Materials and Methods. Western blot analysis was performed to detect the subcellular localization of NF-*κ*B using an antibody against the NF-*κ*B subunit p65. *β*-actin was used as a cytosolic marker and fibrillarin served as a nuclear marker.

We observed an increase in ROS production after LPS stimulation at the indicated dose (Figure 1(a)). To detect the effect of LPS on cell viability, the WST-1 was used in indicated concentrations (0, 1, 3, and 10 *μ*g/mL) of LPS-treated HK-2 cells. Results demonstrated that the cell viability was not changed even in the highest concentration (10 *μ*g/mL) of LPS (Figure 1(b)). The JNK pathway is activated by stress and inflammatory signals [24]. To investigate the inflammatory effect of LPS on HK-2 cells, we evaluated JNK phosphorylated and NF-*κ*B nuclear translocation by Western blot analysis. The result obtained indicated that LPS-induced JNK phosphorylation (JNK pathway activation) without alerting in the total JNK protein levels (Figures 2(a) and 2(b)) and induced the nuclear translocation of NF-*κ*B p65 (Figure 3, lane 6).

### 3.2. LPS-Induced RAW 264.7 Cell Chemotaxis

**Figure 4:**
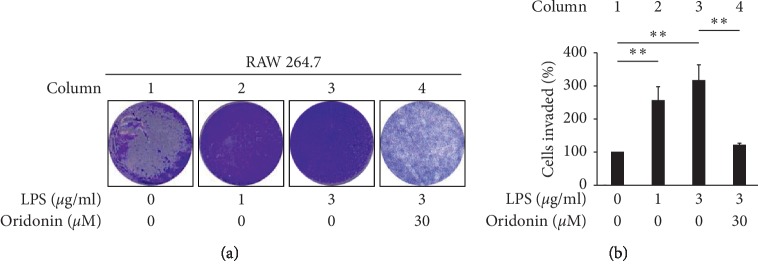
Effects of LPS on the recruitment of monocyte/macrophage RAW 264.7 cells with or without oridonin treatment. HK-2 cells with or without LPS treatment were assayed for their ability to recruit monocyte/macrophage cells (RAW 264.7) in the presence or absence of oridonin. The details are described in Materials and Methods. (a) Macroscopic observation of Transwell chambers (upper). (b) Relative levels of migrated cells presented as the extent of crystal violet staining measured at OD570. All data are presented as mean ± SD. *n* = 3. ^*∗∗*^*p* < 0.01.

To confirm the inflammatory effects caused by LPS, we devised an *in vitro* microenvironment to examine macrophage chemotaxis following LPS stimulation of HK-2 cells. We observed that groups treated with 1, 3, or 10 *μ*g/mL LPS (Figures 4(a) and 4(b), column 2, 3) showed a greater than twofold increase in macrophage chemotaxis as compared with the untreated group (Figures 4(a) and 4(b), column 1).

### 3.3. Oridonin Attenuated LPS-Induced HK-2 Inflammation

**Figure 5:**
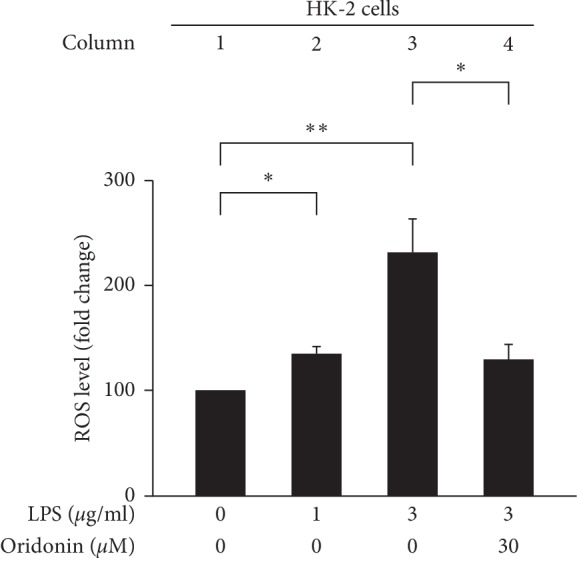
Oridonin attenuated ROS accumulation in LPS-treated HK-2 cells. HK-2 cells were pretreated with oridonin for 12 h before the addition of LPS. The levels of intracellular ROS were determined using DCF-DA, and the fluorescence was detected using FACSCalibur analysis. All data are presented as mean ± SD. *n* = 3. ^*∗*^*p* < 0.05, ^*∗∗*^*p* < 0.01.

**Figure 6:**
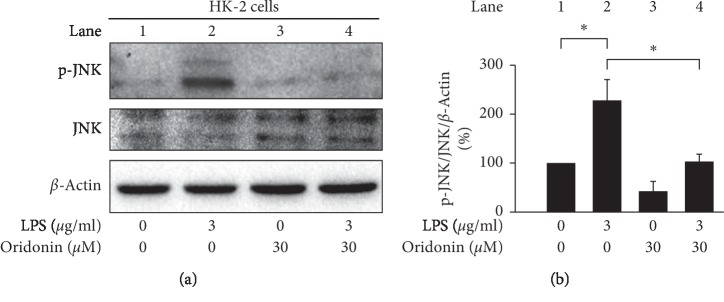
Oridonin attenuated LPS-induced phosphorylation of JNK. (a) HK-2 cells were pretreated with oridonin (30 *μ*M) for 12 h before the addition of 3 *μ*g/mL LPS. Phosphorylation of JNK was analyzed by immunoblotting using antibodies against phosphorylated and total protein. (b) Densitometric analysis of all samples normalized against the level of total protein. All data are presented as mean ± SD. *n* = 3. ^*∗*^*p* < 0.05.

We evaluated the effects of oridonin on LPS-induced ROS production, JNK phosphorylation, NF-*κ*B nuclear translocation in HK-2 cells, and RAW 264.7 chemotaxis. The results obtained indicated that oridonin treatment attenuated LPS-induced ROS production (Figure 5, column 3, 4), JNK phosphorylation (Figure 6, lanes 2, 4), and nuclear translocation of NF-*κ*B p65 (Figure 3, lanes 6, 8). Oridonin also inhibited LPS-induced RAW 264.7 cells chemotaxis (Figure 4, columns 3, 4).

**Figure 7:**
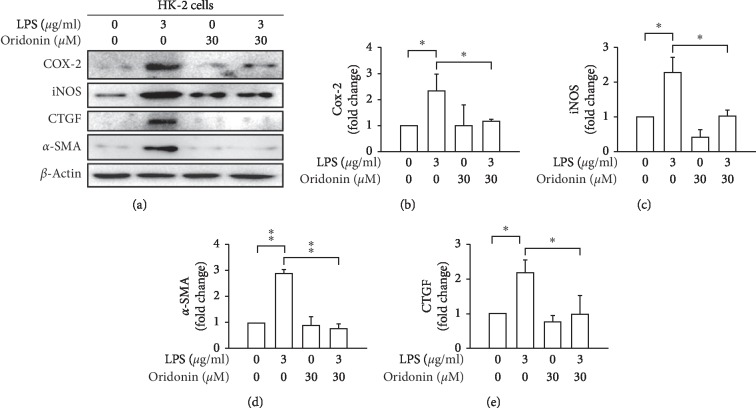
Oridonin attenuated LPS-induced iNOS, COX-2, *a*-SMA, and CTGF expression. HK-2 cells were pretreated with oridonin (30 *μ*M) for 12 h before the addition of 3 *μ*g/mL LPS. iNOS, COX-2, *a*-SMA, and CTGF were analyzed by immunoblotting. (b-e) Densitometric analysis of all samples normalized against the level of total protein. All data are presented as mean ± SD. *n* = 3. ^*∗*^*p* < 0.05, ^*∗∗*^*p* < 0.01.

To further confirm the anti-inflammatory effect of oridonin on LPS-treated HK-2 cells, we evaluated the expressions of iNOS and Cox-2 by Western blotting. Results demonstrated that oridonin treatment attenuated LPS-induced increasing in iNOS and Cox-2. These findings supported that oridonin has an anti-inflammatory effect on LPS-induced HK-2 cell inflammation *in vitro* (Figure 7).

### 3.4. Oridonin Attenuated LPS-Induced HK-2 Fibrotic Effect

To further confirm the antifibrotic-related effect of oridonin on LPS-treated HK-2 cells, we evaluated the expressions of CTGF and *a*-SMA by Western blotting. Results demonstrated that oridonin treatment attenuated LPS-induced increasing in CTGF and *a*-SMA. These findings supported that oridonin has an antifibrotic effect on LPS-induced renal fibrosis *in vitro* (Figure 7).

## 4. Discussion

In the present study, oridonin preconditioning exhibited protective effects on LPS-induced iNOS expression, ROS accumulation, JNK phosphorylation, and NF-*κ*B nuclear translocation, COX-2, *a*-SMA, and CTGF expression in HK-2 cells. Oridonin also inhibited RAW 264.7 chemotaxis. To the best of our knowledge, this is the first study to explore the role of oridonin in LPS-induced ROS accumulation, inflammation, and fibrotic effect in HK-2 cells and RAW 264.7 cell chemotaxis.

Several studies have proposed that the JNK, p38, and extracellular signal regulated kinase (ERK) signaling pathways are involved in LPS-induced inflammatory cytokine production [24, 25]. Activation of JNK signaling is a common phenomenon in most forms of human kidney injury, evident in both intrinsic glomerular and tubular cells as well as in infiltrating leukocytes. Similar patterns of JNK activation are evident in animal models of acute and chronic renal injury [26]. NF-*κ*B nuclear translocation is an indicator of chronic inflammation and ROS accumulation during the progression and development of CKD [27, 28]. Therefore, the inhibition of inflammation and oxidative stress may serve as a good strategy for the treatment of CKD. Su et al. reported the renal protective effect of Fangjifuling against LPS-induced inflammatory and apoptotic responses *in vitro* and *in vivo* [29]. Similar protective effects were reported for berberine against chronic renal failure mediated via tumor necrosis factor receptor associated factor 5- (TRAF5-) induced activation of the NF-*κ*B signaling pathway in mouse podocytes [30]. Consistent with the results of the previous studies [28, 31], we proposed that traditional Chinese medicine regularly used in clinical patients may provide protective effects against kidney injury.

Oridonin exerts protective effects against diabetes-induced renal injury via the Toll-like receptor 4 (TLR4)/*p*38-mitogen-activated protein kinase (MAPK) and NF-*κ*B signaling pathways [32]. Furthermore, oridonin enhanced 5-fluorouracil-triggered 786-O cell necroptosis via the ROS-dependent but JNK-, p38-, and ERK-independent, cascades. This effect suggests its potential application in combination with chemotherapeutic agents for cancer treatment [33, 34].

Macrophage activation depends on the surrounding microenvironment and heterogeneous cell populations that are present in all tissues. Tumor-associated macrophages (TAMs) may be divided into classically activated inflammatory macrophages (M1) and alternatively activated anti-inflammatory macrophages (M2) [35]. LPS has been reported to activate M1 macrophages via NF-*κ*B nuclear translocation [36]. Consistent with our findings (Figure 4), it has been reported that the 4-O-methylhonokiol analog GS12021 inhibits TNF-*α* inflammation and macrophage chemotaxis [37]. We propose that oridonin may exert protective effects on macrophage infiltration. Hence, the anti-inflammation effects may contribute to alleviating RAW 264.7 chemotaxis. Our results suggest that oridonin treatment may serve as a feasible treatment strategy for kidney injury, particularly concerning inflammatory responses.

The inflammatory response is closely associated with JNK phosphorylation activated by cytokines, nutrients, growth factors, and physical-chemical-mechanical stress [38]. Therefore, inhibition of JNK by targeting myeloid cells with a drug may provide therapeutic benefits for the treatment of inflammation-related liver diseases [39]. In the present study, the results indicated that oridonin attenuated LPS-induced HK-2 cells JNK phosphorylation (Figure 6, lane 2, 4), NF-κB activation (NF-*κ*B nuclear translocation, Figure 3 lane 6, 8), and ROS accumulation (Figure 5, column 3, 4) in HK-2 cells. We propose that inflammation is a systemic response dependent on various signaling pathways, consistent with a previous study by Chen et al. [40].

**Figure 8:**
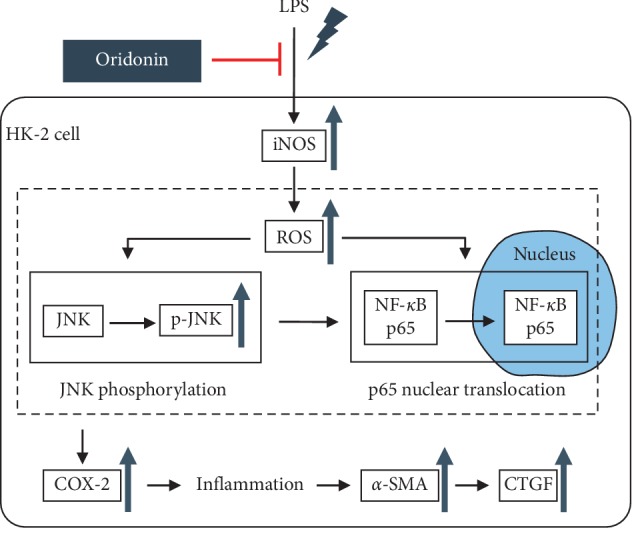
Anti-inflammation and antifibrotic mechanisms of oridonin. Oridonin preconditioning exhibited protective effects on LPS-induced iNOS expression, ROS accumulation, JNK phosphorylation, and NF-*κ*B nuclear translocation, COX-2, *a*-SMA, and CTGF expression in HK-2 cells.

**Figure 9:**
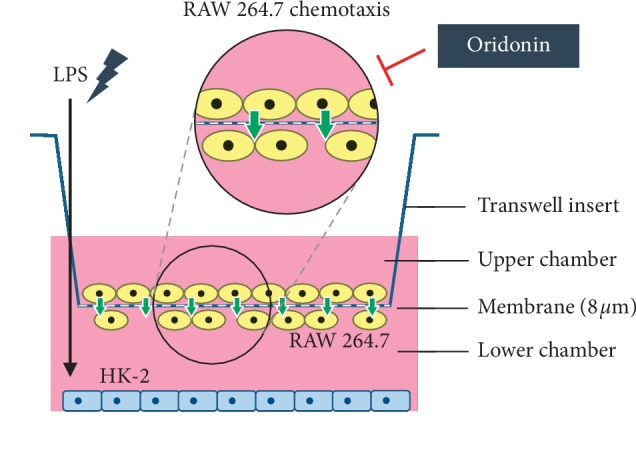
Antichemotaxis mechanisms of oridonin. Oridonin inhibited RAW 264.7 cell chemotaxis.

In summary, our results demonstrated that oridonin preconditioning exhibited protective effects on LPS-induced iNOS expression, ROS accumulation, JNK phosphorylation, and NF-*κ*B nuclear translocation, COX-2, *a*-SMA, and CTGF expression in HK-2 cells (Figure 8). Oridonin also inhibited RAW 264.7 chemotaxis (Figure 9). These novel findings should deepen our understanding of the mechanistic action of oridonin. Since oridonin has been proven to attenuate LPS-induced inflammation in HK-2 cells and inhibit RAW 264.7 cell chemotaxis, further *in vivo* study based on Vhlh conditional knockout mice to mimic chronic kidney inflammation in our previous study [23] is required to determine the potential effects of oridonin on human renal proximal tubular epithelial cells in individuals with CKD.

## Data Availability

The original data used to support the findings of this study are included in the article.
